# Supplementary material for: Electron–acoustic phonon coupling in single crystal CH3NH3PbI3 perovskites revealed by coherent acoustic phonons
Source: Nat Commun. 2017 Feb 8;8:14398. doi: 10.1038/ncomms14398 (PMC5309855; doi:10.1038/ncomms14398)
Supplement: Supplementary Information — Supplementary Figures, Supplementary Notes and Supplementary References [file ncomms14398-s1.pdf]

Electron- Acoustic Phonon Coupling in Single Crystal CH3NH3PbI3 Perovskites Revealed by Coherent Acoustic Phonons

Pierre-Adrien Mante,[1] Constantinos Stoumpos,[2] Mercouri Kanatzidis,[2] and Arkady Yartsev[1]

[1] Department of Chemical Physics and NanoLund, Lund University, Sweden.

[2] Department of Chemistry, Northwestern University, United States.

Supplementary note 1

Due to the different penetration depth of light at different wavelength, the intensity, and thus the energy deposited by the pump light, will have a spatial dependence:

$$I(z,t) = (1-R)I_0 f(t)e^{-\alpha z}$$

Therefore the total stress is proportional to:

$$\sigma(z,t) \propto \left[-3B\beta N\frac{h\nu - E_g}{C_p} - 3B\beta n\frac{E_g - E_t}{C_p} - Nd_e - (N-n)d_h\right](1-R)I_0 f(t)e^{-\alpha z} = \sigma_\lambda I(z,t)$$

Since we are measuring the acoustic signal at the specific Brillouin frequency of 12 GHz in $CH_3NH_3PbI_3$, it is important to characterize the change of the frequency content of the generated acoustic pulse at 12 GHz. The frequency spectrum of the strain is proportional to:

$$\tilde{\eta}(\omega) \propto (1-R)\sigma_\lambda \frac{\omega\tau}{1+\omega^2\tau^2},$$

where $\tau = v/\xi$, with v the longitudinal sound velocity and ξ a characteristic length of the excitation in the system. Depending on the material properties, ξ can represent various physical parameter. In general, it corresponds to the penetration depth of light within the material, however, in the case of strong thermal or charge diffusion, the value of ξ can be lengthened compared to the penetration depth and lead to a changed central frequency of the pulse. This is the case in GaAs where the diffusion is supersonic. In the case of $CH_3NH_3PbI_3$, charge diffusion is much slower, and we can consider the penetration depth as the characteristic length of the excitation.

In Supplementary Figure 1, we reproduced the Fourier spectra of the acoustic pulse for a generation at a pump wavelength of either 550 or 650 nm, assuming a similar value of σ_λ. From

these spectra, we observe that when the penetration depth of light is shorter (550 nm), the content of the spectra is shifted towards higher frequency.

We see that if we want to compare the stress generated at different pump wavelength through the amplitude of the CAP at a specific frequency, we need to consider a correction factor γ=0.8, to account for the change of the phonon frequency spectrum induced by the change of penetration depth.

Supplementary note 2

As we have seen in the manuscript, we can express the stress generated by the absorption of the pump laser beam as follows:

$$\sigma = \sigma_{TE} + \sigma_{DP} = -3B\beta N \frac{h\nu - E_g}{C_p} - 3B\beta n \frac{E_g - E_t}{C_p} - (N-n)d_e - Nd_h \quad \text{(S1).}$$

The stress is directly proportional to the amplitude of the generated coherent acoustic phonons (CAPs). Therefore, if we call α, the ratio between the amplitude in Fig. 3, we then have taking into account the change of the frequency spectrum due to the different pump absorption:

$$\sigma_{550} = \alpha\gamma\sigma_{650} \quad \text{(S2),}$$

$$\sigma_{TE,550} - \alpha\gamma\sigma_{TE,650} = (\alpha\gamma - 1)\sigma_{DP} \quad \text{(S3),}$$

$$3(\alpha\gamma - 1)B\beta\left(N\frac{h\nu_{650} - E_g}{C_p} + n\frac{E_g - E_t}{C_p}\right) - 3\alpha\gamma B\beta N\frac{h\nu_{550} - h\nu_{650}}{C_p} = (\alpha\gamma - 1)[(N-n)d_e - Nd_h] \quad \text{(S4).}$$

As a first approximation, we can neglect the contribution from trap states to the thermoelastic generation, as the additional energy from these traps $(E_g - E_t) \approx 0.08$ eV is much smaller than the energy from relaxation in the conduction band $h\nu_{650} - E_g$. We used a heat capacity of 200 $J.K^{-1}\cdot mol^{-1}$, [S1] a linear thermal coefficient 1.32×10^{-4} K^{-1}, [S2] a band gap energy of 1.55 eV that we determined experimentally from the reflectivity spectrum and a bulk modulus of 12.2 GPa. [S3] We were then able to estimate the stress originating from deformation potential $\sigma_{DP} = (N-n)d_e - Nd_h$

As we mentioned previously, the stress is directly proportional to the amplitude of the generated CAPs. However, we cannot directly estimate the reflectivity change induced by the stress. We know that, taking into account the correction factor due to the different absorption, for the same photoexcited carrier density, the difference in amplitude for different pump wavelengths is solely due to the change of energy of the pump photons:

$$I_{550} - \gamma I_{650} = -3\alpha B\beta N\frac{h\nu_{550} - h\nu_{650}}{C_p} \quad \text{(S5).}$$

We can now estimate the reflectivity change induced by the stress, and furthermore, since we know σ_{DP}, we can then calculate σ_{TE} .

If we now perform experiments with the same pump and probe conditions on both samples, the discrepancy in the amplitude of generated CAPs is directly related to the different trap states:

$$\sigma_{TE1} - \sigma_{TE2} = -3B\beta n(1-\varepsilon)\frac{E_g - E_t}{C_p} \quad \text{(S6)},$$

$$\sigma_{DP1} - \sigma_{DP2} = -n(1-\varepsilon)d_e \quad \text{(S7)},$$

with ε, the ratio of defects between the two samples that we estimated to be 1.5 by monitoring the scattering rate of coherent acoustic phonons. Using Eq. S6, we can thus extract the density of traps, *n*. Then using Eq. S7, we obtain the conduction band deformation potential d_e, and subsequently the valence band deformation potential.

Supplementary note 3

To verify the reliability of our method to obtain the deformation potential, we applied similar approach to a material with well-known properties, GaAs. We performed experiments with pump wavelengths of 550 and 650 nm as depicted in supplementary figure 2.

First of all, we notice that the period of oscillations is much shorter than for $CH_3NH_3PbI_3$. This is due to the larger sound velocity in GaAs. We then performed the analysis to estimate the deformation potential. However, in the case of GaAs, electron diffusion is more important, and electrons that participate in the generation are thus spread on a longer distance than the penetration depth. In that case, the spectrum of the strain due to deformation potential mechanism can be written: [4]

$$\widetilde{U}(\omega) \propto \frac{(1-R)d}{h\nu\rho c^3}\frac{(-i\omega)^2 m_D\omega_D}{\omega_D(\omega_R - i\omega)+\omega^2}\left[\frac{\omega_D}{\omega^2 + m_D^2\omega_D^2} + \frac{1}{\sqrt{\omega_R - i\omega}\left(m_D\omega_D^{1/2} + \sqrt{\omega_R - i\omega}\right)}\right]$$

In the case of the thermoelastic generation, electrons quickly lose their energy to the lattice, on a sub-picosecond time scale, and we can then neglect the contribution of diffusion. The characteristic length of the thermoelastic generation is thus the penetration depth of the pump light, similar to the $CH_3NH_3PbI_3$ case.

We thus obtain the following correction factors γ_{DP}=0.87 and γ_{TE}=0.78, for the deformation potential and the thermoelastic part of the strain, respectively. After calculation, we estimate the deformation potential as d_e+d_h=-10.9 eV, which is close to the literature values. [S5]

Supplementary Figure 1

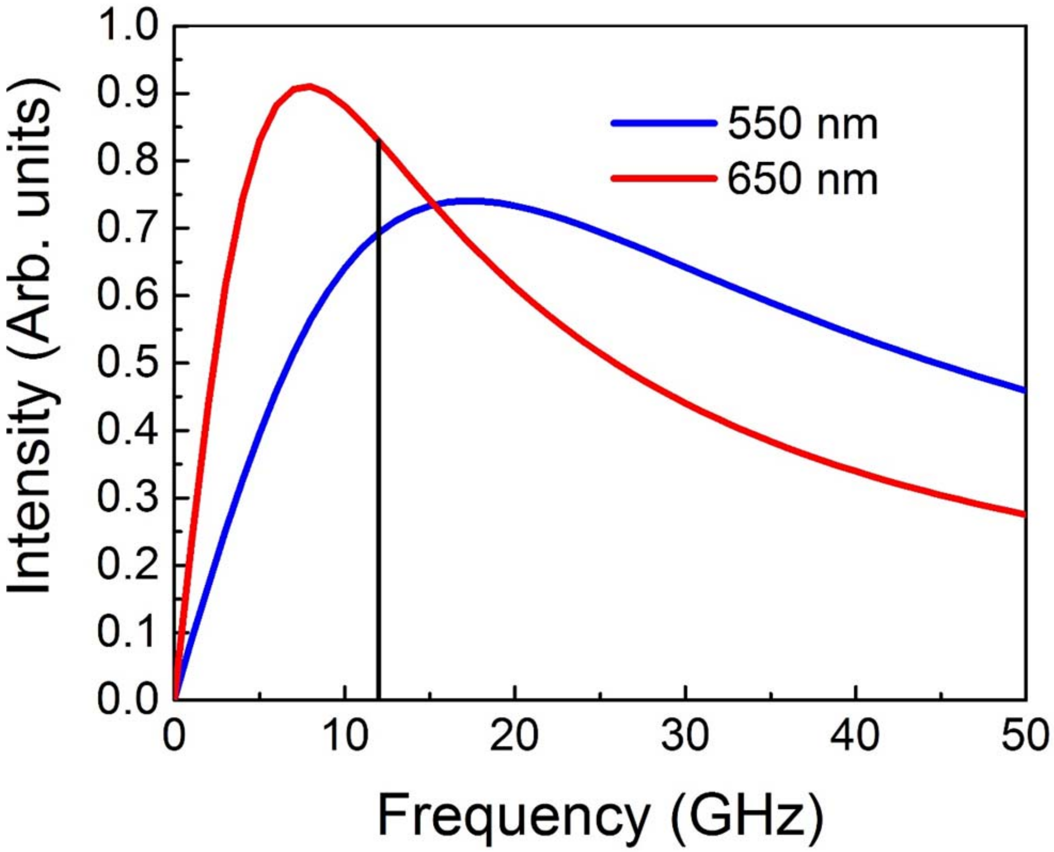

82

83 **Wavelength dependent acoustic phonon frequency spectrum.** Spectrum of coherent acoustic phonons generated in CH3NH3PbI3 for a pump wavelength of either 550 or 650 nm

85 **Supplementary Figure 2**

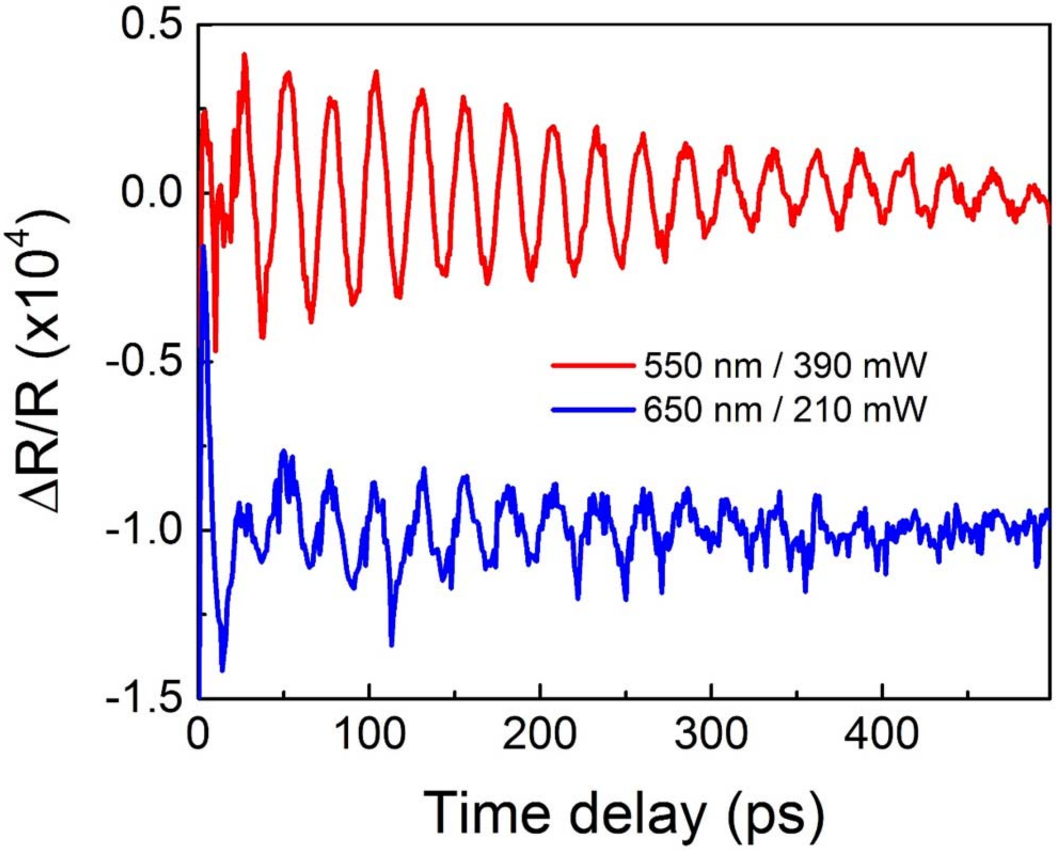

Coherent phonons in GaAs. Transient reflectivity signals at a constant probe wavelength of 850 nm, and a pump wavelength of either 550 or 650 nm of GaAs.

Supplementary Figure 3

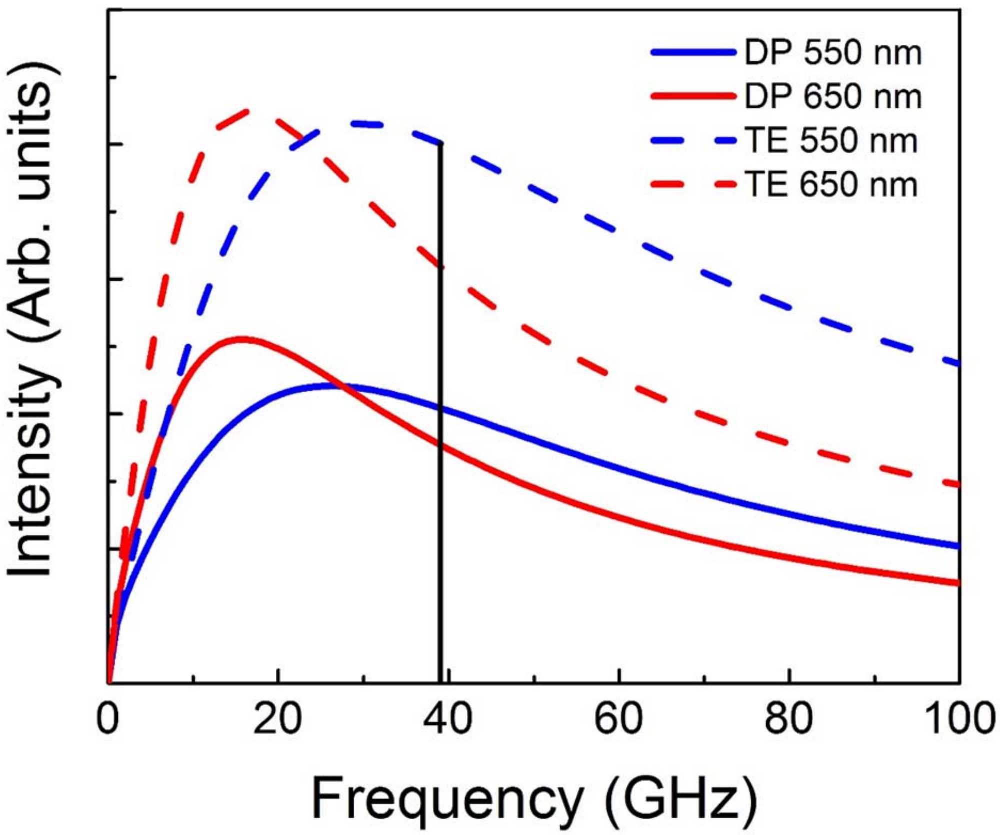

Wavelength dependent acoustic phonon frequency spectrum in GaAs. Spectrum of coherent acoustic phonons generated by deformation potential mechanism or thermoelastcity in GaAs for a pump wavelength of either 550 or 650 nm

References:

[S1] O. Knop, R, E. Wasylishen, M. A. White, T. S. Cameron, M. J. M. Van Oort, *Can. J. Chem*. 1990, **68**, 412.

[S2] T. J. Jacobsson, L. J. Schwan, M. Ottosson, A. Hagfeldt, T. Edvinsson, *Inorg. Chem*. 2015, **54**, 10678.

[S3] J. Feng, *APL Materials* 2014, **2**, 081801.

[S4] S. A. Akhmanov and V. E. Gusev, Usp. Fiz. Nauk 162, 3 (1992)

[S5] P. Ruello, V. Gusev, Ultrasonics 2015, 56, 21
